# Crystal structures of crotonaldehyde semicarbazone and crotonaldehyde thiosemicarbazone from X-ray powder diffraction data

**DOI:** 10.1107/S2056989015000663

**Published:** 2015-01-17

**Authors:** Atef Arfan, Mwaffak Rukiah

**Affiliations:** a Department of Chemistry, Atomic Energy Commission of Syria (AECS), PO Box 6091, Damascus, Syrian Arab Republic

**Keywords:** crystal structure, crotonaldehyde, semicarbazone, thio­semicarbazone, powder X-ray diffraction, supra­molecular structure, hydrogen bond, one-dimensional chain, two-dimensional networks

## Abstract

Crotonaldehyde semicarbazone {systematic name: (*E*)-2-[(*E*)-but-2-en-1-yl­idene]hydrazinecarboxamide}, C_5_H_9_N_3_O, (I), and crotonaldehyde thio­semi­carba­zone {systematic name: (*E*)-2-[(*E*)-but-2-en-1-yldene]hydra­zinecarbo­­thio­amide}, C_5_H_9_N_3_S, (II), show the same *E* conformation around the imine C=N bond. Compounds (I) and (II) were obtained by the condensation of crotonaldehyde with semicarbazide hydro­chloride and thio­semicarbazide, respectively. Each mol­ecule has an intra­molecular N—H⋯N hydrogen bond, which generates an *S*(5) ring. In (I), the crotonaldehyde fragment is twisted by 2.59 (5)° from the semicarbazide mean plane, while in (II) the corresponding angle (with the thio­semicarbazide mean plane) is 9.12 (5)°. The crystal packing is different in the two compounds: in (I) inter­molecular N—H⋯O hydrogen bonds link the mol­ecules into layers parallel to the *bc* plane, while weak inter­molecular N—H⋯S hydrogen bonds in (II) link the mol­ecules into chains propagating in [110].

## Abstract 2

Crotonaldehyde semicarbazone and crotonaldehyde thio­semicarbazone show the same *E* conformation around the imine C=N bond. Each mol­ecule has an intra­molecular N—H⋯N hydrogen bond, which generates an *S*(5) ring. Inter­molecular N—H⋯O hydrogen bonds in the semicarbazone link the mol­ecules into layers parallel to the *bc* plane, while weak inter­molecular N—H⋯S hydrogen bonds in the thio­semicarbazone link the mol­ecules into chains propagating in [110].

## Chemical context   

The chemistry of semicarbazones and thio­semicarbazones is especially inter­esting due to their special role in biological applications such as anti-proliferative, anti-tumoral, anti-convulsant, anti-trypanosomal, herbicidal and biocidal activities (Beraldo *et al.*, 2002 ▸; Kasuga *et al.*, 2003 ▸; Teixeira *et al.*, 2003 ▸; Beraldo & Gambino, 2004 ▸; Mikhaleva *et al.*, 2008 ▸; de Oliveira *et al.*, 2008 ▸; Alomar *et al.*, 2012 ▸; Gan *et al.*, 2014 ▸). They are also important inter­mediates in organic synthesis, mainly for obtaining heterocyclic rings, such as thia­zolidones, oxa­diazo­les, pyrazolidones, and thia­diazo­les (Greenbaum *et al.*, 2004 ▸; Küçükgüzel *et al.*, 2006 ▸). Semicarbazones and thio­semicarbazones have received considerable attention in view of their simplicity of preparation, various complexing abilities and coordination behavior that can be used in analytical applications (Garg & Jain, 1988 ▸; Casas *et al.*, 2000 ▸). They are of inter­est from a supra­molecular point of view since they can be functionalized to give different supra­molecular arrays.
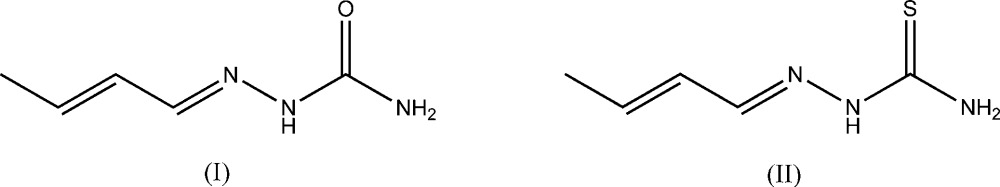


## Structural commentary   

**Figure 1:**
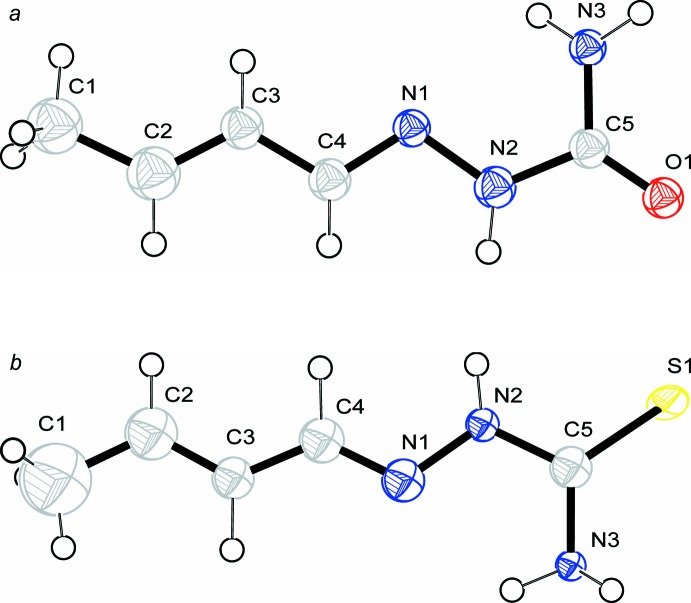
The mol­ecular structures of (*a*) (I) and (*b*) (II), showing the atom-labelling schemes. Displacement spheres (and the ellipsoid for S1) are drawn at the 50% probability level.

Compounds (I) and (II) crystallize in centrosymmetric space groups *P2_1_/c* and *P* 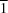, respectively, with one mol­ecule in the asymmetric unit. Each mol­ecule has an intra­molecular N—H⋯N hydrogen bond (Tables 1 ▸ and 2 ▸), which forms an *S*(5) ring. The semicarbazone and thio­semicarbazone fragments in the compounds show an *E* conformation around the imine C=N bond. The mol­ecules (Fig. 1 ▸) are approximately planar, with a dihedral angle of 2.59 (5)° between the C1/C2/C3 crotonaldehyde plane and the mean plane of the C4/N1/N2/C5/O1/N3 semicarbazone fragment for (I), and of 9.12 (5)° between the C1/C2/C3 crotonaldehyde plane and the mean plane of the C4/N1/N2/C5/S1/N3 thio­semicarbazone fragment for (II). All bond lengths and angles in (I) and (II) are normal and correspond well to those observed in the crystal structures of related semi- and thio­semicarbazone derivatives, *viz*. acetone semicarbazone and benzaldehyde­semicarbazone (Naik & Palenik, 1974 ▸), 3,4- methyl­ene­dioxy­benzaldehyde­semicarbazone (Wang *et al.*, 2004 ▸), isatin 3-semicarbazone and 1-methyl­isatin 3-semicarbazone (Pelosi *et al.*, 2005 ▸), 4- (methyl­sulfan­yl)benzaldehyde­thio­semicarbazone (Yathirajan *et al.*, 2006 ▸), 4-(methyl­sulfan­yl)benzaldehyde­semicarbazone (Sarojini *et al.*, 2007 ▸), 5-hy­droxy-2-nitro­benzaldehyde thio­semicarbazone (Reddy *et al.*, 2014 ▸) and 1-(4-formyl­benzyl­idene) thio­semicarbazone (Carballo *et al.*, 2014 ▸).

## Supra­molecular features   

**Figure 2:**
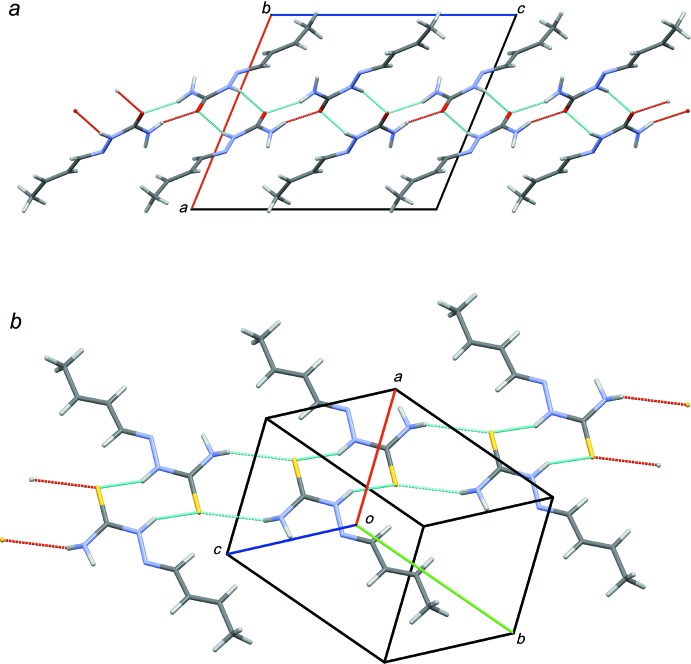
(*a*) A portion of the crystal packing of (I) viewed down the *b* axis (parallel to the hydrogen-bonded layer). (*b*) A portion of the crystal packing of (II), showing the hydrogen-bonded chain of the mol­ecules. Thin dotted lines denote inter­molecular hydrogen bonds.

As a result of the presence of potential hydrogen-donor sites in mol­ecules (I) and (II), supra­molecular hydrogen-bonding inter­actions are observed in both compounds (Tables 1 ▸ and 2 ▸). In the crystal of (I), mol­ecules are linked by pairs of N—H⋯O hydrogen bonds, forming inversion dimers with *R^2^_2_(8)* ring motifs (Fig. 2 ▸ *a*). The resulting dimers are connected through N—H⋯O hydrogen bonds, forming layers parallel to *bc* plane. In the crystal of (II), mol­ecules are linked by weak N—H⋯S hydrogen bonds, forming chains propagating in [110] (Fig. 2 ▸ *b*).

## Synthesis and crystallization   

All reactions and manipulations were carried out in air with reagent grade solvents. The IR spectra were recorded on a Jasco FT–IR 300E instrument. ^1^H and ^13^C{^1^H} NMR spectra were recorded on a Bruker Bio spin 400 spectrometer. Microanalysis was performed using EURO EA. Powder X-ray diffraction data were collected with Stoe Transmission diffractometer (Stadi P).

For the synthesis of (I), a mixture of semicarbazide hydro­chloride (CH_5_N_3_O·HCl; 0.5 g, 4.5 mmol) and sodium acetate (CH_3_COONa; 0.75 g, 9.1 mmol) in 10 ml water was agitated well and crotonaldehyde (0.5 g, 7.1 mmol) was added slowly with stirring. On completion of the addition, the reaction mixture was agitated for 24 h at room temperature. The solid product which formed was separated by filtration and washed with water and finally recrystallized from absolute ethanol to produce the product (I) (white powder; m.p. 481–482 K) in 55.5% yield.

IR (KBr, ν, cm^−1^): 3456, 3281, 3192 (NH_2_), (1668–1638) (C=O); ^1^H NMR (400 MHz, CD_3_OD) δ p.p.m. 1.76 (*d*, *J* = 4.42 Hz, 3H, –CH_3_), 6.43–5.46 (*m*, 2H, –HC=CH–), 7.39 (*d*, *J* = 7.19 Hz, 1H, HC=N–).^13^C NMR (100 MHz, CD_3_OD) δ p.p.m. 18.52 (CH_3_), 130.01 (–HC=CH–), 137.62 (–HC=CH–), 145.64 (N=C), 160.19 (C=O). Analysis calculated for (I): C, 47.23; H, 7.13; N, 33.05, 12.58 O%. Found: C, 46.43; H, 6.08; N, 34.69%

For the synthesis of (II), crotonaldehyde (0.5 g, 7.1 mmol) was added to thio­semicarbazide (CH_5_N_3_S; 0.65 g, 7.1 mmol) in 5 ml water and the mixture was stirred at room temperature for 24 h. The product was separated by filtration and recrystallized from absolute ethanol to produce the product (II) (white powder; m.p. 435–436 K) in 72.5% yield.

IR (KBr, ν, cm^−1^): 3323, 3244, 3164 (NH_2_), 1650(C=S). ^1^H NMR (400 MHz, CDCl_3_) δ p.p.m. 1.90 (*d*, *J =* 5.86 Hz, 3H, –CH_3_), 6.07–6.27 (*m*, 2H, –HC=CH–), 6.49 (*sb*, 1H), 7.10 (*sb*, 1H) 7.60 (*d*, *J =* 8.57 Hz, 1H, HC=N–), 10.10 (*sb*, 2H). ^13^C NMR (100.6 MHz, CDCl_3_) 18.73 (CH_3_), 127.70 (–HC=CH–), 140.58 (–HC=CH–), 146.21 (N=C), 177.95 (C=S). Analysis calculated for (II): C, 41.93; H, 6.33; N, 29.34.05, 22.39 S%. Found: C, 41.89; H, 6.25; N, 31.88%.

## Refinement details   

Crystal data, data collection and structure refinement details are summarized in Table 3 ▸. Compounds (I) and (II) crystallized in the form of a very fine white powder. Since no single crystals of sufficient size and quality could be obtained, the crystal structures of both compounds were determined from X-ray powder diffraction patterns. The powder samples of (I) and (II) were lightly ground in a mortar, loaded into two Mylar foils and fixed onto the sample holder with a mask of suitable inter­nal diameter (8.0 mm). The powder X-ray diffraction data were collected at room temperature with a STOE transmission STADI-P diffractometer using monochromatic Cu *K_a_*_1_ radiation (λ= 1.54060 Å) selected with an incident beam curved-crystal germanium Ge(111) monochromator with a linear position-sensitive detector (PSD). The patterns were scanned over the angular range 5.0–80.0° (2θ). For pattern indexing, the extraction of the peak positions was carried out with the program *WinPLOTR* (Roisnel & Rodríguez-Carvajal, 2000 ▸). Pattern indexing was performed with the program *DICVOL4.0* (Boultif & Louër, 2004 ▸). The first 20 lines of the powder pattern were indexed completely on the basis of a monoclinic cell for (I) and a triclinic cell for (II). The figures of merit (de Wolff *et al.*, 1968 ▸; Smith & Snyder, 1979 ▸) are sufficiently acceptable to support the obtained indexing results [*M*(20) = 50.5, *F*(20) = 71.9 (0.0034, 83)] for (I) and [*M*(20) = 61.8, *F*(20) = 96.0 (0.0051, 41)] for (II). The best estimated space groups were *P2_1_/c* in the monoclinic system for (I) and *P* 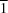 in the triclinic system for (II).

The whole powder diffraction patterns from 5 to 80° (2θ) for the two compounds (I) and (II) were subsequently refined with cell and resolution constraints (Le Bail *et al.*, 1988 ▸) using the profile-matching option of the program *FULLPROF* (Rodríguez-Carvajal, 2001 ▸). The number of mol­ecules per unit cell was estimated to be *Z* = 4 for (I) and *Z* = 2 for (II). The initial crystal structures for (I) and (II) were determined by direct methods using the program *EXPO2014* (Altomare *et al.*, 2013 ▸). The models found by this program were introduced into the program *GSAS* (Larson & Von Dreele, 2004 ▸) implemented in *EXPGUI* (Toby, 2001 ▸) for Rietveld refinement. During the Rietveld refinements, the background was refined using a shifted Chebyshev polynomial with 20 coefficients. The effect of asymmetry of low-order peaks was corrected using a pseudo-Voigt description of the peak shape (Thompson *et al.*, 1987 ▸), which allows for angle-dependent asymmetry with axial divergence (Finger *et al.*, 1994 ▸) and microstrain broadening, as described by Stephens (1999 ▸). The two asymmetry parameters of this function, *S*/*L* and *D*/*L*, were both fixed at 0.022 during this refinement. Intensities were corrected from absorption effects with a function for a plate sample in transmission geometry with a μ·*d* value of 0.15 for (I) and 0.72 for (II) (μ is the absorption coefficient and *d* is the sample thickness). These μ·*d* values were determined experimentally.

**Figure 3:**
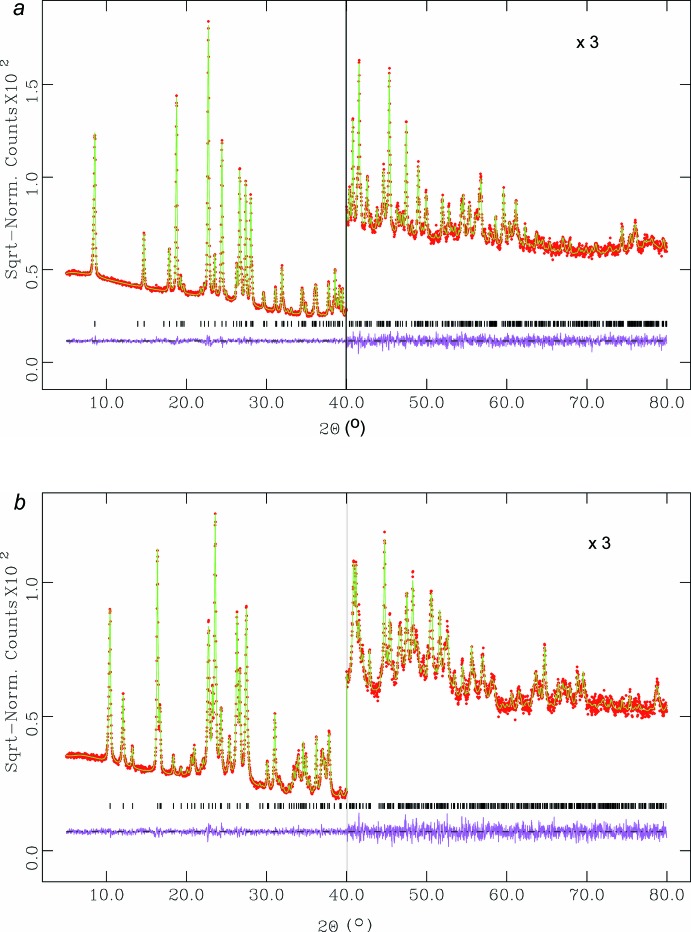
The final Rietveld plots for (*a*) (I) and (*b*) (II). Experimental intensities are indicated by dots and the best-fit profile (upper trace) and difference pattern (lower trace) are shown as solid lines. The vertical bars indicate the calculated positions of the Bragg peaks.

Before the final refinement, all H atoms were introduced in geometrically calculated positions. The coordinates of these H atoms were refined with strict restraints on bond lengths and angles until a suitable geometry was obtained, after that they were fixed in the final stage of the refinement. No soft restraints were imposed for (I), while for (II) the CH_3_—CH bond was clearly stretched (close to 1.6 Å), therefore a single soft restraint was carried out to obtain a normal value (1.49 Å). The final refinement cycles were performed using isotropic atomic displacement parameters for the C, N and O atoms, an anisotropic atomic displacement parameter for S atom in (II) and a fixed global isotropic atomic displacement parameter for the H atoms. The preferred orientation was modelled with 12 coefficients using a spherical harmonics correction (Von Dreele, 1997 ▸) of intensities in the final refinement. The use of the preferred orientation correction leads to a better mol­ecular geometry with better agreement factors. The final Rietveld plots of the X-ray diffraction patterns for both (I) and (II) are given in Fig. 3 ▸.

## Supplementary Material

Crystal structure: contains datablock(s) CROTON-CZ_Publ, I, II. DOI: 10.1107/S2056989015000663/cv5481sup1.cif

Rietveld powder data: contains datablock(s) I. DOI: 10.1107/S2056989015000663/cv5481Isup2.rtv

Rietveld powder data: contains datablock(s) II. DOI: 10.1107/S2056989015000663/cv5481IIsup3.rtv

CCDC references: 1043290, 1043289

Additional supporting information:  crystallographic information; 3D view; checkCIF report

## Figures and Tables

**Table 1:** Hydrogen-bond geometry (, ) for (I)

*D*H*A*	*D*H	H*A*	*D* *A*	*D*H*A*
N3H2N3N1	0.87	2.33	2.629(19)	100
N2H1N2O1^i^	0.88	2.07	2.910(11)	158
N3H1N3O1^ii^	0.91	2.04	2.914(18)	162

Symmetry codes: (i) 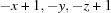; (ii) 
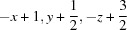
.

**Table 2:** Hydrogen-bond geometry (, ) for (II)

*D*H*A*	*D*H	H*A*	*D* *A*	*D*H*A*
N3H2N3N1	0.89	2.17	2.641(14)	112
N2H1N2S1^i^	0.86	2.83	3.473(11)	133
N3H1N3S1^ii^	0.87	2.77	3.398(11)	130

Symmetry codes: (i) 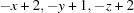; (ii) 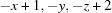.

**Table 3:** Experimental details

	(I)	(II)
Crystal data		
Chemical formula	C_5_H_9_N_3_O	C_5_H_9_N_3_S
*M*_r_	127.15	143.21
Crystal system, space group	Monoclinic, *P*2_1_/*c*	Triclinic, *P* 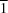
Temperature (K)	298	298
*a*, *b*, *c* ()	11.1646(3), 5.13891(9), 13.0301(2)	5.86650(17), 8.0313(2), 9.0795(4)
, , ()	90, 112.3496(11), 90	104.1407(18), 101.0403(19), 106.3511(17)
*V* (^3^)	691.43(3)	382.15(2)
*Z*	4	2
Radiation type	Cu *K*_1_, = 1.5406	Cu *K*_1_, = 1.5406
(mm^1^)	0.74	3.11
Specimen shape, size (mm)	Flat sheet, 8 8	Flat sheet, 8 8

Data collection		
Diffractometer	Stoe transmission Stadi-P	Stoe transmission Stadi-P
Specimen mounting	Powder loaded into two Mylar foils	Powder loaded into two Mylar foils
Data collection mode	Transmission	Transmission
Scan method	Step	Step
2 values ()	2_min_ = 5 2_max_ = 80 2_step_ = 0.02	2_min_ = 4.980 2_max_ = 79.960 2_step_ = 0.02

Refinement		
*R* factors and goodness of fit	*R*_p_ = 0.027, *R*_wp_ = 0.036, *R*_exp_ = 0.029, *R*(*F* ^2^) = 0.02795, ^2^ = 1.613	*R*_p_ = 0.033, *R*_wp_ = 0.043, *R*_exp_ = 0.034, *R*(*F* ^2^) = 0.02670, ^2^ = 1.664
No. of data points	3750	3750
No. of parameters	121	114
No. of restraints	0	1
H-atom treatment	H-atom parameters not refined	H-atom parameters not refined

Computer programs: *WinXPOW* (Stoe Cie, 1999 ▸), *EXPO2014* (Altomare *et al.*, 2013 ▸), *GSAS* (Larson Von Dreele, 2004 ▸), *ORTEP-3 for Windows* (Farrugia, 2012 ▸), *Mercury* (Macrae *et al.*, 2006 ▸) and *publCIF* (Westrip, 2010 ▸).

## supplementary crystallographic information

### Crystal data

**Table:**

C_5_H_9_N_3_S	*V* = 382.15 (2) Å^3^
*M**_r_* = 143.21	*Z* = 2
Triclinic, *P*1	*F*(000) = 152
Hall symbol: -P 1	*D*_x_ = 1.245 Mg m^−^^3^
*a* = 5.86650 (17) Å	Cu *K*α_1_ radiation, λ = 1.5406 Å
*b* = 8.0313 (2) Å	µ = 3.11 mm^−^^1^
*c* = 9.0795 (4) Å	*T* = 298 K
α = 104.1407 (18)°	Particle morphology: fine powder
β = 101.0403 (19)°	white
γ = 106.3511 (17)°	flat sheet, 8 × 8 mm

### Data collection

**Table:**

Stoe transmission Stadi-P diffractometer	Data collection mode: transmission
Radiation source: sealed X-ray tube	Scan method: step
Ge 111 monochromator	2θ_min_ = 4.980°, 2θ_max_ = 79.960°, 2θ_step_ = 0.02°
Specimen mounting: Powder loaded into two Mylar foils	

### Refinement

**Table:**

Least-squares matrix: full	Profile function: CW Profile function number 4 with 21 terms Pseudovoigt profile coefficients as parameterized in (Thompson *et al.*, 1987) Asymmetry correction of Finger *et al.*, 1994. #1(GU) = 0.000 #2(GV) = 0.000 #3(GW) = 2.793 #4(GP) = 0.000 #5(LX) = 5.477 #6(ptec) = 2.45 #7(trns) = 0.00 #8(shft) = 0.0000 #9(sfec) = 0.00 #10(S/L) = 0.0220 #11(H/L) = 0.0220 #12(eta) = 0.6000 Peak tails are ignored where the intensity is below 0.0010 times the peak Aniso. broadening axis 0.0 0.0 1.0
*R*_p_ = 0.033	114 parameters
*R*_wp_ = 0.043	1 restraint
*R*_exp_ = 0.034	H-atom parameters not refined
*R*(*F*^2^) = 0.02670	(Δ/σ)_max_ = 0.03
χ^2^ = 1.664	Background function: GSAS Background function number 1 with 20 terms. Shifted Chebyshev function of 1st kind 1: 590.360 2: -469.557 3: 198.126 4: -45.2586 5: -2.75624 6: 13.8508 7: 4.35563 8: -5.95029 9: -12.8815 10: 35.6051 11: -12.9276 12: -11.1488 13: 8.85293 14: -2.01034 15: -0.496121 16: 8.39616 17: -2.33367 18: -5.14527 19: 10.5079 20: -3.85249
3750 data points	

### Fractional atomic coordinates and isotropic or equivalent isotropic displacement parameters (Å^2^)

**Table:**

	*x*	*y*	*z*	*U*_iso_*/*U*_eq_	
C1	0.184 (2)	0.841 (2)	0.515 (2)	0.103 (6)*	
H1A	0.15342	0.79934	0.39748	0.12*	
H1B	0.2323	0.97016	0.55142	0.12*	
H1C	0.02574	0.78525	0.53491	0.12*	
C2	0.370 (2)	0.7688 (17)	0.5865 (18)	0.054 (5)*	
H2	0.53963	0.83335	0.59455	0.055*	
C3	0.325 (2)	0.6393 (16)	0.6524 (15)	0.034 (5)*	
H3	0.14582	0.56255	0.63049	0.055*	
C4	0.487 (3)	0.5747 (19)	0.7264 (19)	0.039 (5)*	
H4	0.66632	0.65671	0.74816	0.055*	
N1	0.4514 (17)	0.4461 (12)	0.7878 (15)	0.035 (4)*	
N2	0.6486 (16)	0.4005 (12)	0.8462 (13)	0.021 (4)*	
H1n2	0.79218	0.45838	0.841	0.05*	
C5	0.611 (3)	0.2572 (16)	0.907 (2)	0.034 (4)*	
N3	0.3681 (15)	0.1560 (12)	0.8849 (13)	0.017 (4)*	
H1n3	0.34725	0.13246	0.97116	0.05*	
H2n3	0.26401	0.20773	0.84645	0.05*	
S1	0.8446 (6)	0.1980 (5)	0.9772 (6)	0.04081	

### Atomic displacement parameters (Å^2^)

**Table:**

	*U*^11^	*U*^22^	*U*^33^	*U*^12^	*U*^13^	*U*^23^
S1	0.032 (4)	0.039 (5)	0.082 (8)	0.026 (4)	0.043 (5)	0.035 (5)

### Geometric parameters (Å, º)

**Table:**

C1—H1A	0.999	C4—N1	1.274 (12)
C1—H1B	0.946	N1—N2	1.361 (10)
C1—H1C	0.983	N2—H1n2	0.856
C1—C2	1.49 (2)	N2—C5	1.377 (13)
C2—H2	0.963	C5—N3	1.376 (13)
C2—C3	1.311 (13)	C5—S1	1.638 (13)
C3—H3	1.008	N3—C5	1.376 (13)
C3—C4	1.352 (14)	N3—H1n3	0.872
C4—H4	1.024	N3—H2n3	0.894
			
H1A—C1—H1B	109.0	C3—C4—N1	130.8 (16)
H1A—C1—H1C	106.2	H4—C4—N1	116.8
H1A—C1—C2	108.5	C4—N1—N2	118.6 (11)
H1B—C1—H1C	110.2	N1—N2—H1n2	119.6
H1B—C1—C2	113.8	N1—N2—C5	119.2 (10)
H1C—C1—C2	108.8	H1n2—N2—C5	121.2
C1—C2—H2	115.8	N2—C5—N3	115.6 (12)
C1—C2—C3	125.6 (13)	N2—C5—S1	120.4 (11)
H2—C2—C3	118.2	N3—C5—S1	123.5 (9)
C2—C3—H3	116.8	C5—N3—H1n3	110.5
C2—C3—C4	128.4 (15)	C5—N3—H2n3	112.2
H3—C3—C4	113.9	H1n3—N3—H2n3	113.2
C3—C4—H4	111.8		
			
C4—N1—N2—C5	−177.4 (14)	N1—N2—C5—N3	8.0 (19)
N2—N1—C4—C3	175.6 (15)	C1—C2—C3—C4	−176.2 (15)
N1—N2—C5—S1	179.6 (11)	C2—C3—C4—N1	−177.6 (16)

### Hydrogen-bond geometry (Å, º)

**Table:**

*D*—H···*A*	*D*—H	H···*A*	*D*···*A*	*D*—H···*A*
N3—H2N3···N1	0.89	2.17	2.641 (14)	112
N2—H1N2···S1^i^	0.86	2.83	3.473 (11)	133
N3—H1N3···S1^ii^	0.87	2.77	3.398 (11)	130

Symmetry codes: (i) −*x*+2, −*y*+1, −*z*+2; (ii) −*x*+1, −*y*, −*z*+2.
